# A Translation Initiation Element Specific to mRNAs with Very Short 5′UTR that Also Regulates Transcription

**DOI:** 10.1371/journal.pone.0003094

**Published:** 2008-08-28

**Authors:** Rofa Elfakess, Rivka Dikstein

**Affiliations:** Department of Biological Chemistry, The Weizmann Institute of Science, Rehovot, Israel; Lehigh University, United States of America

## Abstract

Transcription is controlled by cis regulatory elements, which if localized downstream to the transcriptional start site (TSS), in the 5′UTR, could influence translation as well. However presently there is little evidence for such composite regulatory elements. We have identified by computational analysis an abundant element located downstream to the TSS up to position +30, which controls both transcription and translation. This element has an invariable ATG sequence, which serves as the translation initiation codon in 64% of the genes bearing it. In these genes the initiating AUG is preceded by an extremely short 5′UTR. We show that translation *in vitro* and *in vivo* is initiated exclusively from the AUG of this motif, and that the AUG flanking sequences create a strong translation initiation context. This motif is distinguished from the well-known Kozak in its unique ability to direct efficient and accurate translation initiation from mRNAs with a very short 5′UTR. We therefore named it TISU for Translation Initiator of Short 5′UTR. Interestingly, this translation initiation element is also an essential transcription regulatory element of Yin Yang 1. Our characterization of a common transcription and translation element points to a link between mammalian transcription and translation initiation.

## Introduction

In prokaryotes translation of mRNA is coupled to transcription whereas in eukaryotes it takes place in the cytosolic compartment, separated by the nuclear membrane from the transcriptionally active chromosomes. The eukaryotic translation process has thus been considered to be entirely independent of transcription.

Transcription of protein-encoding genes is controlled by two types of DNA elements, enhancer and core promoter. The gene specific enhancer elements serve as binding sites for transcription regulatory factors and can be divided into two classes: those that function independently of their position relative to the transcription start site (TSS) and those that can activate transcription only when located proximal to the TSS. The core promoter is situated around the TSS and is the site on which RNA polymerase II and general transcription factors (GTFs) assemble into a pre-initiation complex. Specific combinations of different regulatory elements determine a unique transcriptional control program for each gene. Some of these transcription regulatory elements are localized downstream to the TSS and are present in the mRNA as well, often in the 5′UTR, so they are also in a position where the could influence the translation stage of gene expression. However presently there is little evidence for such composite regulatory elements. In this study we identified an abundant proximal element with a strict location near and downstream to the TSS. It serves as a translation initiation element optimized to facilitate translation from genes with an extremely short 5′UTR, and, in addition, it proved to be a functional Ying Yang 1 (YY1) transcription regulatory element. Our findings suggest that this type of regulatory elements may provide a link between transcription and post-transcriptional stages of gene expression.

## Results

### Identification of a translation initiation element in mRNAs with short 5′UTR

**Figure 1:**
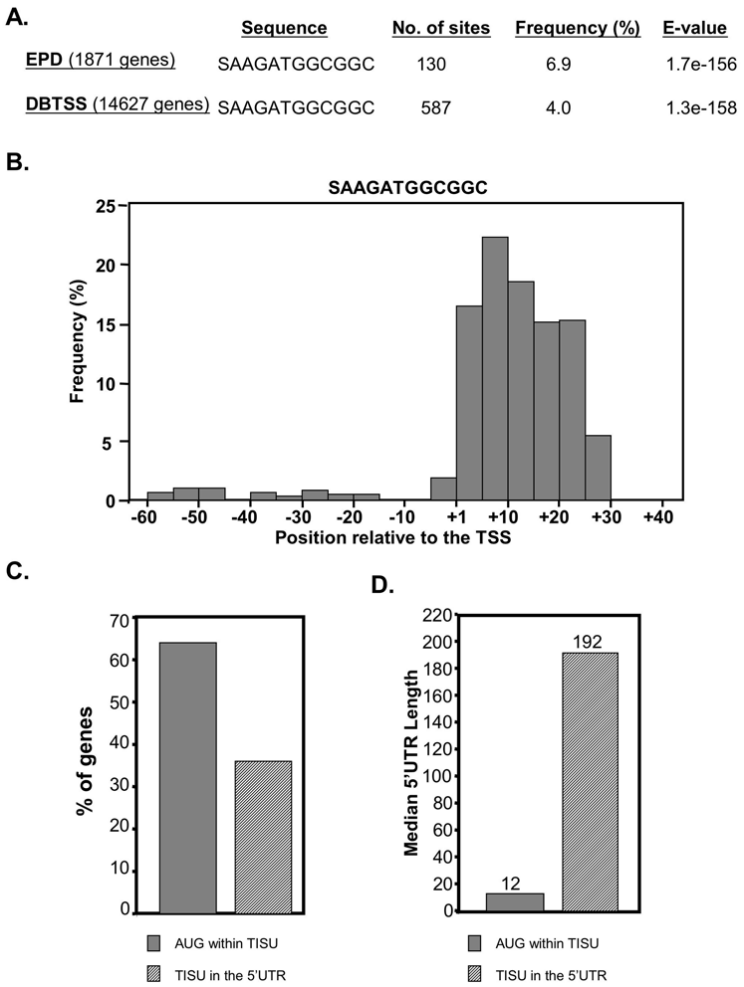
The sequence and features of the TISU motif. A. TISU element was identified by analyzing 1871 (EPD) and 14,628 (DBTSS) human proximal promoter sequences, from −60 to +40 relative to the TSS, with the MEME program. For each dataset the identified sequence, the number of sites, the frequency and the E-values are shown. The E-value refers to the probability that a motif of the same width is found with equally or higher likelihood in the same number of random sequences having the same single-nucleotide frequencies as the promoter set. B. The distribution of TISU at 5 nt intervals throughout the proximal promoter region (−60 to +40 relative to the TSS) as determined by the DBTSS. C. Analysis of TISU as a translation initiation element. Genes containing TISU were divided into two groups, those in which translation starts within TISU (gray bars) and those in which translation starts downstream of TISU (striped bars). The graph shows the percentage of genes from each group. D. The median length (in nt) of the 5′UTR in the two groups.

We retrieved promoter sequences with verified TSSs from the EPD and the DBTSS (1871 and 14,628 genes respectively), and searched for motifs that are overrepresented in the −60 to +40 region relative to the TSS by the MEME program [1] that looks for conserved un-gapped blocks in a set of query sequences, and was set to return motifs of 6–12 nucleotides long. A highly significant motif emerged from both databases (Fig. 1A). The frequency of the motif in the proximal promoter region among human genes is ∼4% (587 genes). We determined the distribution of this motif relative to the TSS (Fig. 1B) and found that it is restricted to downstream positions, from +5 up to +30. The motif was identified at the same location by another computational study [2], but its functional significance was not analyzed. Functional classification of the motif-containing genes revealed statistically significant enrichment in fundamental cellular activities such as protein biogenesis and degradation, protein folding, RNA metabolism and mitochondrial functions (Table 1).

**Table 1:** Functional classification of genes bearing the TISU element downstream to the TSS as determined by the Database for Annotation, Visualization and Integrated Discovery (DAVID).

Cluster	Enrichment	Term	P-value
1	15.41	Protein biosynthesis (Ribosom &Translation)	6.25E-30
1	15.41	Mitochondrion	2.66E-23
1	15.41	Protein folding	1.33E-07
2	12.59	RNA metabolism	3.51E-33
2	12.59	RNA processing	2.08E-28
3	11.52	Ubiquitin cycle	3.88E-23

The table describes clusters with the highest enrichment score within the gene list and the most significant terms within each cluster with their P-value.

Using the same MEME program we analyzed the upstream and downstream sequences that flank the −60 to +40 region in order to assess whether this motif is unique to the proximal promoter region. Neither the upstream nor downstream flanking sequences of the −60 to +40 region were enriched with this element whereas the CAAT box and Sp1, which are known upstream promoter elements, and the downstream Kozak translation initiation sequence, were identified (data not shown).

Close inspection of the motif's sequence revealed a high degree of invariability in the core sequence AAGATGGC, particularly the central ATG triplet. Taking into account that the motif is present in the 5′UTR we reasoned that its ATG might also serve as a translation initiation codon. To test this possibility the mRNA sequences of the 554 genes containing the motif in downstream position, were retrieved from the UCSC Genome Browser (http://genome.ucsc.edu/) and analyzed for their translation initiation site as specified by the database. The results revealed that the open reading frame of the majority of genes containing the motif (64%) begin from its ATG (Fig. 1C).

As the motif is located very close to the transcription initiation site but not further downstream, the 5′UTR length in the genes in which this element comprises the translation initiation site is extremely short with a median value of 12 nucleotides (Fig. 1D). On the other hand the median 5′UTR length in the 36% of genes in which this element does not comprise the translation initiation site, is 192 nucleotides (Fig. 1D), which is close to the median 5′UTR length of mammalian mRNAs (150 nucleotides, [3]). Thus this element represents a translation initiation context characteristic of genes with a very short 5′UTR. We named this motif TISU for Translation Initiator of Short 5′UTR (see below).

### TISU is an important transcriptional regulatory element

**Figure 2:**
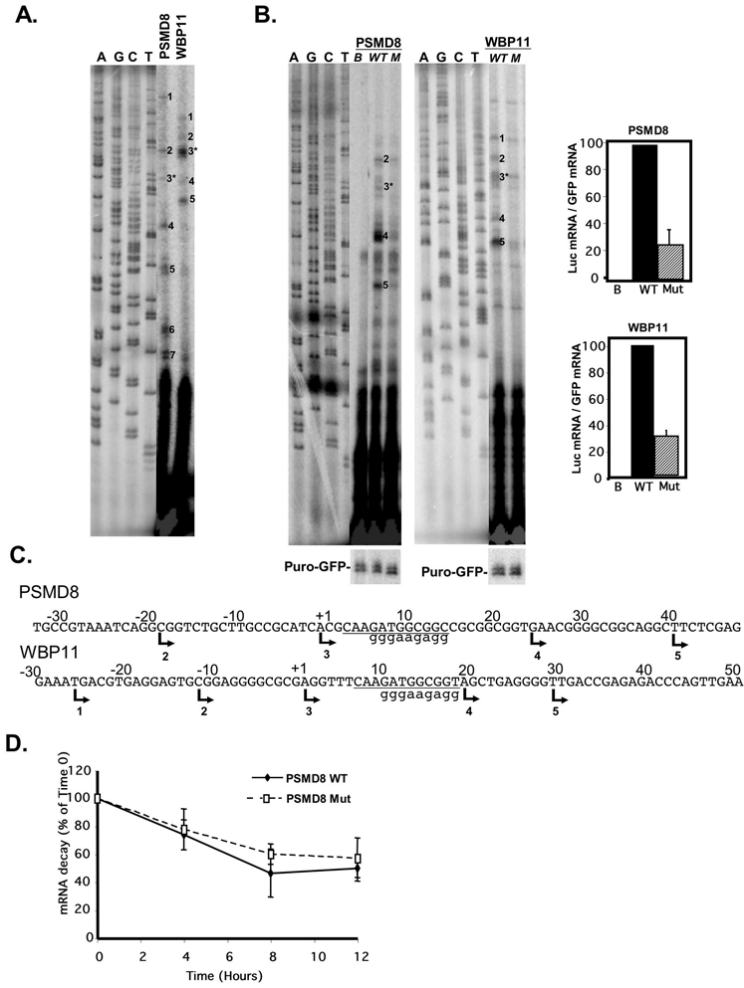
TISU is essential for transcription directed by PSMD8 and WBP11 promoters. A. Determination, by primer extension, of the transcription start sites of the endogenous PSMD8 and WBP11 genes using gene specific primers as probes and total RNA prepared from 293T cells. The primer-extension products were run together with sequencing reactions (marked A, C, G and T). The TSSs are numbered and their positions are shown in panel C. B. The effect of TISU mutation on transcription. The promoters of the PSMD8 and WBP11 genes (from −180 to +50 and −150 to +50 of PSMD8 and WBP11 respectively) were cloned in front of a luciferase reporter gene and then subjected to site directed mutagenesis to create TISU mutants. The wild type (WT) or mutated (M or Mut) promoter or the promoter-less parental plasmid (pGL2-basic, B) was co-transfected into 293T cells with CMV-puro-GFP that serves as a reference for transfection efficiency. 24 hours post transfection RNA was extracted and analyzed by primer extension using luciferase and puromycin primers. The primer-extension products were run together with sequencing reactions (marked A, C, G and T). The TSSs were numbered according to the endogenous TSSs shown in A. The graphs on the right show quantification by densitometry of the TSSs that correspond to the endogenous ones from 3 independent experiments (average ±SD). C. The DNA sequences and the positions of TSSs of the PSMD8 and WBP11 promoters. The TSSs are indicated by arrows and numbers correspond to the numbered TSS bands shown in A&B. An asterisk marks the TSS assigned by the database. Lower case letters indicate the sequence of the TISU mutation. D. The effect of TISU mutation on mRNA stability. Wild type (WT) and TISU-mutated PSMD8 (Mut) luciferase reporter genes were transfected into 293T cells. 24 hours after transfection, transcription was halted by actinomycin D and RNA extracted at different time intervals. To measure the decay of the luciferase mRNA, semi-quantitative PCR was applied using a 5′ primer containing either the wild type or mutated TISU sequence and a luciferase primer as 3′ primer. The results shown are the average ±SD of 4 independent experiments.

Given the proximity of TISU to the TSS we first examined the possibility that it acts as a transcriptional element on two selected genes in which it occurs, PSMD8 and WBP11. First we performed primer extension assays to determine their transcription start sites using primers corresponding to +109 and +122 of PSMD8 and WBP11 respectively, relative to the TSS specified in the database. Each of these genes showed multiple TSSs located upstream and downstream to TISU (Fig. 2A and C). Next, the promoters of these genes (from −180 to +50 and −150 to +50 of PSMD8 and WBP11, respectively) were cloned in front of a luciferase reporter gene. The promoters were then subjected to site directed mutagenesis to create TISU mutants. The wild type (WT) or mutated (Mut) promoter was co-transfected into 293T cells with CMV-puro-GFP that serves as a reference for transfection efficiency. 24 hours post transfection RNA was extracted and analyzed by primer extension using luciferase and puromycin primers. As shown in Fig. 2B the two promoters displayed significant promoter activity (WT lanes) compared to the promoter-less control construct (B lane). Both PSMD8 and WBP11 promoters produced multiple transcription initiation sites, most of them corresponding exactly to the endogenous TSSs (Fig. 2A), with some differences in the relative intensities. For example TSSs 4 and 5 of PSMD8 are stronger in the heterologous than the endogenous context, and the major TSS 3 of endogenous WBP11 is weaker in the heterologous context. The mutation in TISU substantially decreased the relative amount of all the relevant TSSs in both promoters. These results suggest that TISU is important for transcription.

Since some of the TSSs lie upstream to TISU so that its sequence occurs in their 5′UTR the possibility raises that in these transcripts TISU may affect mRNA stability rather than transcription. We therefore determined the rate of mRNA decay in wild type and TISU-mutated PSMD8 luciferase reporter genes transfected into 293T cells. Twenty-four hours after transfection, transcription was halted by actinomycin D and RNA was extracted at different time intervals. To measure specifically the decay of the luciferase mRNA containing TISU or its mutant, RT-PCR was applied using 5′ primers containing either the wild type or mutant TISU sequence and luciferase as the 3′ primer. As shown in Fig. 2D the wild type and TISU mutated transcripts have similar rates of turnover. These results, together with the effect of TISU mutation on TSSs in which TISU is not present in the 5′UTR, confirm that TISU primarily affects transcription of all major TSSs and rule out the possibility that TISU acts to increase mRNA stability.

### TISU is a potent translation initiation element

**Figure 3:**
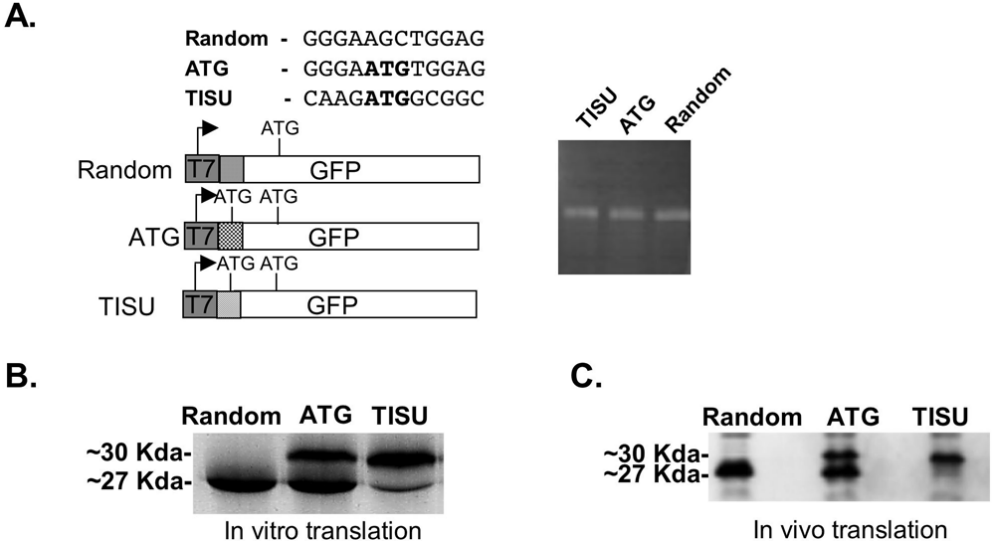
TISU is a strong translation initiation element. A. Schematic representation of the GFP constructs containing a random sequence (random), an upstream ATG within a random context (ATG) or a TISU element (TISU), downstream to the T7 promoter and in frame to the ATG of the GFP (left panel). The constructs were transcribed *in vitro* with the addition of m^7^G followed by DNaseI treatment and visualized by electrophoresis on agarose gel (right panel). B. *In vitro* translation analysis of the capped mRNA. The mRNAs were translated *in vitro* using rabbit reticulocyte extract and ^35^S-met (translation that begins from the original GFP ATG produces a ∼27 Kda protein whereas translation from the upstream inserted ATG generates a ∼30 Kda protein). C. The indicated *in vitro* transcribed and capped mRNAs were transfected into 293T cells and 24 hours later cells were harvested and the translation initiation site was determined by western blot using anti-GFP antibody.

The finding that the open reading frame begins in the ATG of the TISU element in most of the genes bearing it raises the possibility that TISU's sequence may influence translation initiation. To examine its activity as a translational initiation motif we inserted the TISU element downstream to the T7 promoter and upstream to GFP with its ATG in frame with the GFP ATG. An in frame ATG in a random context or a sequence without ATG inserted between the T7 promoter and GFP served as controls (Fig. 3A, upper left panel). These constructs were transcribed and capped *in vitro* with T7 polymerase and treated with DNaseI (Fig. 3A, right panel), and the mRNAs were then translated with rabbit reticulocyte lysate in the presence of ^35^S-methionine. Translation that begins from the original GFP AUG produces a ∼27 Kda protein whereas translation from the upstream inserted AUG is expected to generate a ∼30 Kda protein. As shown in Fig. 3B, translation of the GFP lacking an additional ATG sequence was initiated at the original GFP AUG resulting in a 27 Kda GFP (left lane). The GFP with the AUG in a random context initiated translation from the upstream and more frequently from the downstream AUG (middle lane) whereas the GFP bearing TISU initiated translation mostly from the upstream AUG (right lane).

To examine further the role of TISU in translation initiation, the *in vitro* transcribed GFP mRNAs were transfected into 293T cells and 24 hours later the cells were harvested and subjected to immunoblot using GFP antibody. The results show that in the absence of upstream AUG, GFP was initiated from the original AUG (Fig. 3C, left lane) and in the presence of an upstream AUG in a random context translation was initiated from both the upstream AUG and the original GFP AUG (Fig. 3C, middle lane). By contrast, when the mRNA containing the AUG in the context of TISU was transfected, GFP translation was initiated exclusively from the upstream AUG, with no detectable leakage to the original downstream AUG (Fig. 3C, right lane).

### TISU is a translation initiation element specific for mRNAs with a short 5′UTR

**Figure 4:**
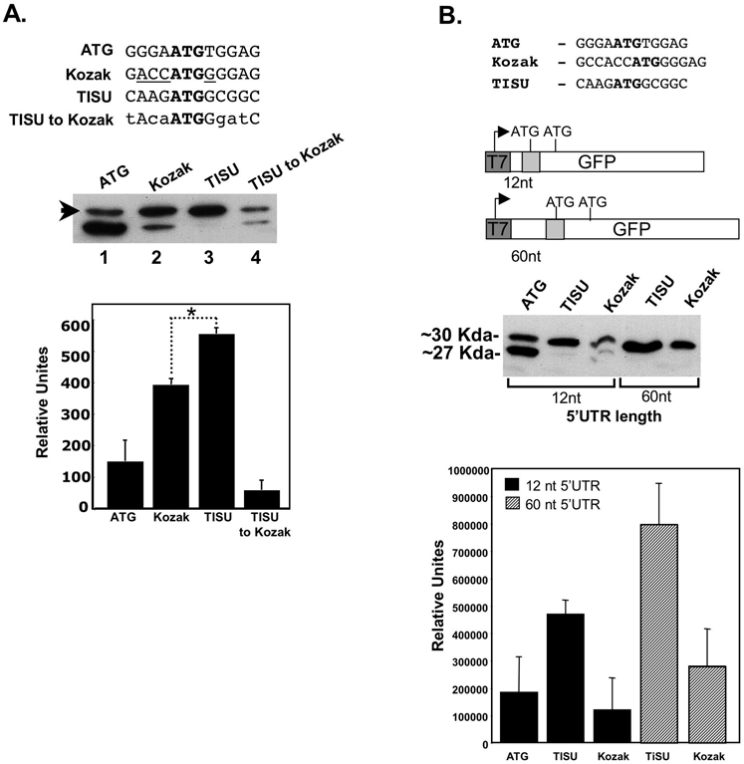
TISU is distinct from the Kozak by its ability to direct accurate translation initiation from very short 5′UTR. A. Upper panel shows the sequences of ATG in a random context, TISU, TISU mutant that was converted into a minimal Kozak (TISU to Kozak), and the Kozak element in the same context as in Fig. 3. Equivalent amounts of *in vitro* transcribed mRNAs were co-transfected into 293T cells together with luciferase mRNA that was used to normalize transfection efficiency. Representative western blot of *in vivo* translation results is shown in the middle panel. The intensity of the accurately initiated product (30 KDa band), indicated by an arrow, was quantified. The results (lower panel) represent the average ±S.D of 3 independent experiments. * p<0.0005. B. A 60 nt linker without ATG was cloned between the T7 promoter and TISU or Kozak sequence (upper panel) in order to increase the length of the 5′UTR from 12 to 60 nt as shown schematically in the middle panel. The constructs with short and long 5′UTR were *in vitro* transcribed and the mRNAs were co-transfected into 293T cells together with luciferase mRNA. 24 h post transfection normalized cell extract was subjected to western blot to determine the translation initiation site. The graph represents densitometric measurements of the intensity of the accurately initiated product (30 KDa band) from two independent experiments.

**Figure 5:**
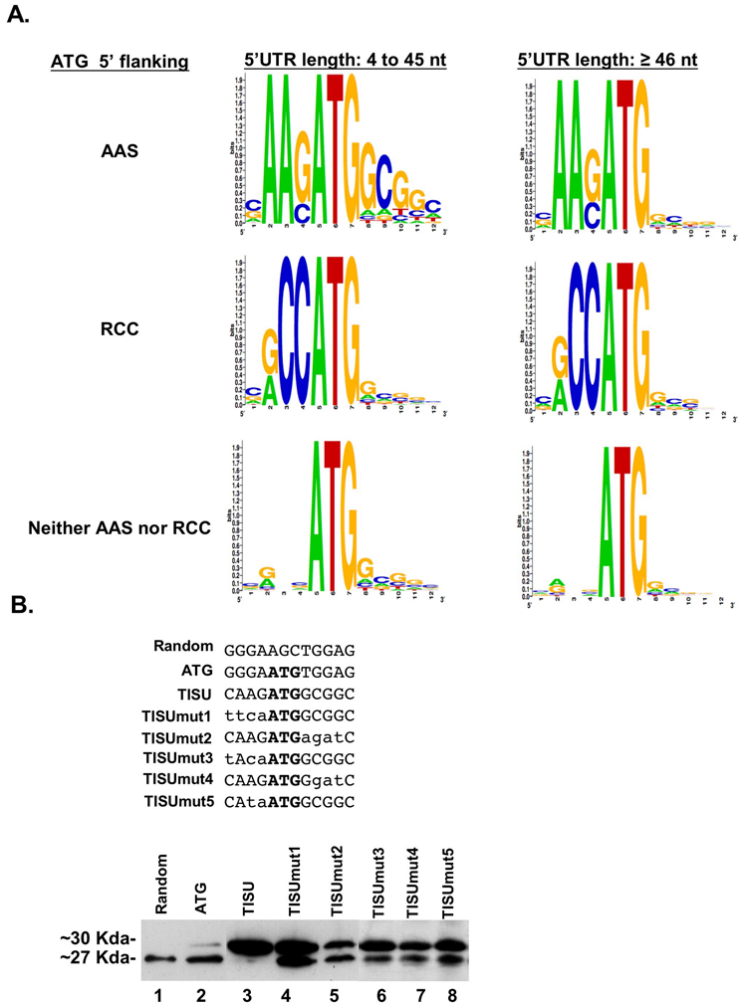
A. TISU is an exclusive translation initiator of genes with short 5′UTR. Genes were divided into 3 groups according to the 5′ flanking sequence of their ORF's AUG (AAS, RCC or neither) and each group was further divided according to their 5′UTR length (short 5′UTR: 4 to 45 nt ; long 5′UTR: ≥46 nt). The frequency of the nucleotides in positions −4 to +8 relative to the AUG of each group is represented in pictogram, where the height of letters corresponds to their frequencies in each position. B. The sequence requirement of the TISU for accurate translation initiation of short 5′UTR mRNAs. The sequences shown in the upper panel were cloned downstream to T7 promoter and in frame with the downstream ATG of GFP. The constructs were transcribed and capped *in vitro*, and the mRNAs were then transfected into 293T cells where they underwent *in vivo* translation, which was analyzed by immunoblot using anti-GFP antibody (lower panel).

The upstream AUG flanking sequence of TISU ( AAGAUGG) deviates somewhat from the Kozak translation initiation consensus ( RCCAUGG) [4]. Previous studies have shown that a purine in the −3 position and a G in the +4 position (the A of the AUG is +1) are sufficient for efficient and accurate translation initiation [5], [6]. Given that TISU has these features we compared its activity either to the full Kozak consensus (Fig. 4A, Kozak) or to a sequence which retained a purine in the −3 and a G in the +4 position while the rest of the flanking sequences were changed (Fig. 4A, upper panel TISU to Kozak). As shown in Fig. 4A (middle panel, lanes 2 and 4) the Kozak and the TISU-to-Kozak sequences have similar translation initiation fidelity as translation was initiated more often from the upstream AUG than the downstream AUG but with a detectable leakage to the downstream AUG. TISU however, directed translation initiation exclusively from the upstream AUG (lane 3) with no detectable leakage to downstream AUG. These results suggest that in addition to the −3 and +4 positions of TISU, sequences in the other positions contribute to its strong translation initiation activity (see also Fig. 5). Quantitative measurements of the motif-directed translation site (the 30 Kda band), using a co-transfected luciferase mRNA as a reference, revealed that the TISU context is stronger than the Kozak or the sequence that conforms to minimal Kozak (Fig. 4A, lower panel). Thus TISU represents an optimal form of translation initiation context.

A previous study using *in vitro* assays had shown that leakiness from a Kozak element to a second downstream AUG occurs when the length of the 5′UTR is shorter than 32 nucleotides [7]. In the experimental set up shown in Fig. 4A, the 5′UTR length is only 12 nucleotides long, yet the fidelity of translation initiation of TISU was very high. As most genes containing TISU have a very short 5′UTR, we examined the effect of the 5′UTR length on translation mediated by the Kozak element and TISU. We synthesized mRNAs in which the distance of the AUG from the cap, in the context of either TISU or the Kozak motifs, was set to be either 12 or 60 nt. In this specific set up we used the extended Kozak consensus (Fig. 4B, upper panel), to rule out the possibility that leakiness to downstream AUG is due to the use of less optimal sequence. Each of these mRNAs was co-transfected together with the luciferase mRNA into 293T cells and GFP protein levels, normalized to luciferase, were analyzed by immunoblot. As shown in Fig. 4B, in the Kozak-containing mRNAs, a detectable leakage to the downstream AUG occurs when the 5′UTR is 12 nt long. Lengthening the 5′UTR however, has a dramatic effect on translation initiation accuracy, eliminating translation from the downstream AUG and increasing translation efficiency. On the other hand, the fidelity of translation in TISU containing mRNAs was similar in the short and long 5′UTR mRNAs. Extending the 5′UTR length in TISU mRNAs resulted in only a small quantitative effect (Fig. 4B lower panel). These findings indicate that the sequence of TISU, in contrast to the Kozak, is optimized to direct efficient and accurate translation initiation from mRNAs which have an extremely short 5′UTR.

### Upstream and downstream AUG flanking sequences of TISU contribute to the high translation fidelity from short 5′UTR

To further analyze TISU as an initiator of translation of mRNAs with a short 5′UTR we examined the context of the initiating AUG in 11,120 human genes. The genes were divided into 3 groups according to the AUG 5′ flanking sequence representing TISU (AAS, S = G or C), Kozak (RCC, R = A or G) and the remaining genes. Each group was then further divided into genes with a short (<46) or long (≥46) 5′UTR. The AUG 3′ flanking sequences in each group were aligned and analyzed by the Weblogo program. This analysis (Fig. 5A) revealed that the AAS 5′ flanking sequence of the initiating AUG is associated with the GCGGC 3′ flanking sequence, reminiscent of TISU, only in mRNAs with a short 5′UTR. On the other hand no differences in the 3′ AUG flanking sequences were observed in short and long 5′UTRs in either Kozak or non-Kozak non-TISU initiating AUGs. The finding provides independent evidence that the 5′ and 3′ AUG flanking sequences of TISU are specific to short 5′UTR mRNAs.

To analyze the sequence requirements of TISU for translation initiation from very short (12 nucleotides) 5′UTR mRNA, we generated mutants upstream and downstream to the initiating AUG (TISU mut1-mut5, Fig. 5B) in the context of the GFP reporter and the T7 promoter. *In vitro* transcribed and capped mRNAs were then transfected into 293T cells and translation initiation of the GFP protein was analyzed by immunoblot. The results (Fig. 5B, lower panel) show that mutation in the 5′ and 3′ flanking sequences reduced translation fidelity and caused significant leakage to the downstream AUG. Of particular interest are mut3, mut4 and mut5 which retain the extended Kozak consensus (RNNAUGG), but nevertheless significantly reduced fidelity. The bioinformatic analysis and the detailed mutagenesis of TISU clearly distinguish it from the Kozak element and further establish it as an initiator of short 5′ UTR mRNAs.

### TISU within a native context drives accurate and efficient translation initiation

**Figure 6:**
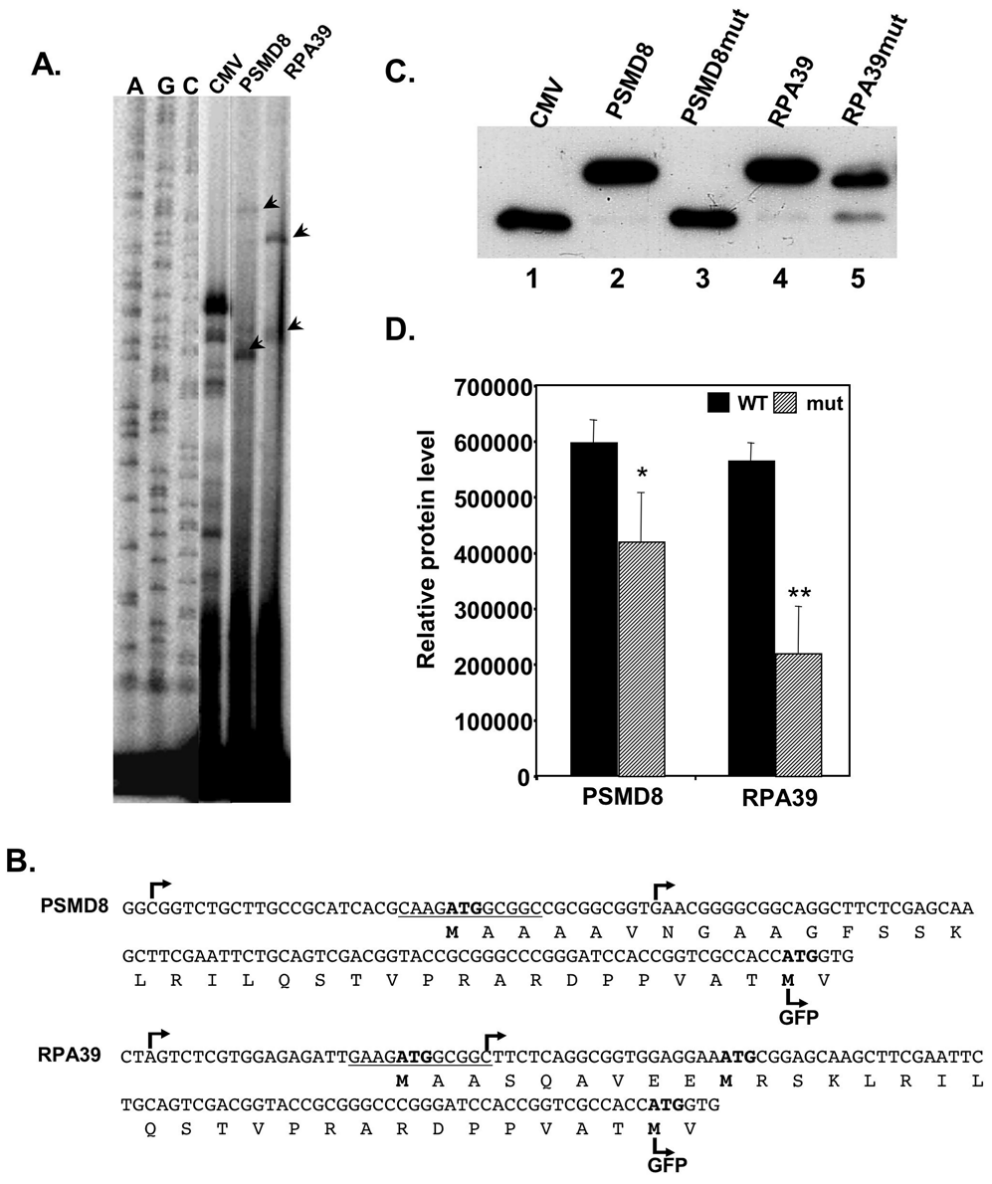
TISU drives translation initiation in a native context. A. PSMD8 and RPA39 promoters and part of the coding sequences were fused in frame to the GFP gene, instead of the CMV promoter. The constructs were transfected into 293T cells and 24 hours post transfection total RNA was extracted and the TSSs, determined by primer extension. The primer-extension products were run together with sequencing ladders (A, C, G, left panel) and the TSSs are indicated by arrowheads. B. The DNA and protein sequences and the positions of TSSs of the PSMD8 and RPA39 sequences. The TSSs are indicated by arrows, the expected translation start sites are marked with bold letters and the translation start site of the GFP is indicated. C. Representative western blot of the translation from mRNA directed by PSMD8 and RPA39 genes. GFP reporter gene driven by PSMD8 and RPA39 promoters bearing wild type and mutated TISU, and the parental CMV promoter were co-transfected into 293T cells together with the luciferase reporter gene pGL3-promoter to normalize transfection efficiency. Normalized cell lysate was subjected to western blot using anti-GFP to determine which AUG initiated translation. D. The intensity of the translation products was quantified. The results represent the average ±S.D of 3 independent experiments. * p<0.05, ** p<0.005.

To examine the activity of TISU in a native context we analyzed translation initiation sites of PSMD8 and RPA39 genes whose open reading frames start within TISU according to the UCSC Genome Browser (the ORF of WBP11 gene analyzed above does not start in TISU and is followed by several stop codons). In RPA39 the TISU ATG precedes an additional in frame ATG located 27 nucleotides downstream (Fig. 6B). The PSMD8 and RPA39 promoters (wild type and TISU mutant) and the beginning of the coding sequences were fused to the green fluorescent protein (GFP) such that the ATG of TISU is in frame with that of the GFP (Fig. 6B). Translation that begins from the original GFP AUG produces a ∼27 Kda protein whereas translation from the upstream TISU AUG of PSMD8 and RPA39 would add 34 and 32 amino acids respectively. These constructs were transfected into 293T cells and 24 hours later cells were harvested. Primer extension analysis confirmed generation of mRNAs that include TISU in the 5′UTR as well as mRNA that initiate downstream to TISU (Fig. 6A and B) as we have shown for the endogenous PSMD8 gene (Fig. 2A). We expect two sites of translation initiation from mRNAs directed from PSMD8 promoter and three sites from RPA39 promoter (see Fig. 6B). Immunoblot analysis with anti-GFP antibody clearly shows that the major GFP protein generated from PSMD8 and RPA39 is larger by ∼3.5 Kda than the parental GFP (Fig. 6C, compare lanes 2 and 4 to lane 1). This indicates that the main site of translation initiation occurred within TISU, and it was substantially more efficient and accurate than translation from the downstream AUGs (lanes 2 and 4). Translation from mRNA transcribed by the PSMD8 mutant promoter (lane 3) was initiated from the downstream GFP AUG. Likewise, translation from the RPA39 mutant promoter (lane 5) was initiated from the native downstream AUG, but in this case there was a significant leakage to the downstream AUG of the GFP. These findings are fully compatible with the *in vivo* translation analysis of TISU in a heterologous context supporting the notion that TISU is a strong translation initiator.

### Characterization of TISU as YY1 transcription regulatory element

**Figure 7:**
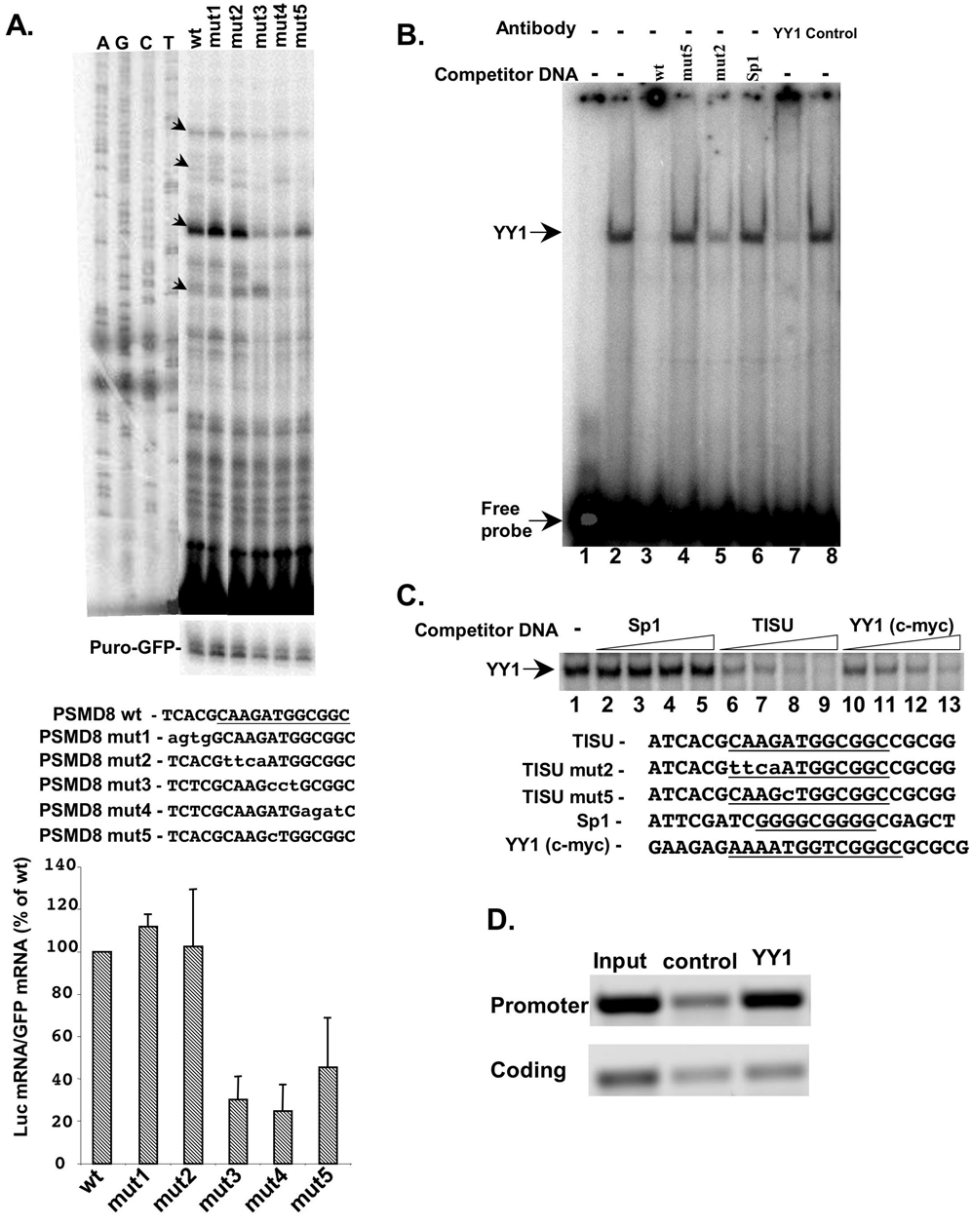
The sequence requirements for the function of TISU as a transcriptional element correlate with the binding of YY1. A. Successive blocks within and outside of TISU (underlined) in the PSMD8 promoter were mutated as shown in the middle panel. The wild type (wt) and mutated constructs were transfected into 293T cells and their mRNA levels analyzed by primer extension and normalized with puro-GFP mRNA. The primer-extension products were run together with sequencing ladders of the wild type construct (A, C, G and T). Representative primer extension assays are shown on the upper panel. Results of densitometric analysis of 3 independent transfection experiments (mean±SD) are shown in the lower panel. The arrows, representing TSSs relevant to the endogenous gene, indicate the quantified bands. B. Electrophoresis mobility shift assay using HeLa cell nuclear extract and a double stranded oligonucleotide containing PSMD8 TISU (from −4 to +19) as a probe. Lane 1, free probe and lane 2, the probe with the nuclear extract. Competitor DNAs were added to the reactions in lanes 3–6 as indicated on the top. The sequences of oligos used for binding and competition are shown in C. In lanes 7 and 8 anti-YY1 and Control IgG were added to the reactions. C. The upper panel shows an EMSA with PSMD8 TISU as a probe and competition with increasing amounts of the sequences indicated on the lower panel. D. YY1 binds to PSMD8 promoter *in vivo*. HeLa cells were subjected to chromatin immunoprecipitation assay using YY1 antibodies and an irrelevant antibodies as a control. Representative PCR analyses of the promoter and the coding region of PSMD8 gene of two independent experiments are shown.

The results shown in Fig. 2 indicate that TISU is also an important transcription regulatory element. Its sequence fits the consensus of the Ying Yang 1 (YY1) binding site, but in this strict downstream location, it appears only in one orientation. To examine in more detail the sequence requirements for TISU to act as a transcriptional element and its relation to YY1, several successive blocks within the motif or upstream to it in the PSMD8 promoter were mutated (Fig. 7A, middle panel, mut2 and mut4 correspond to mut1 and mut2 in Fig. 5B). In addition a single substitution was generated in which the invariable A at position 5 (relative to the beginning of the motif) that corresponds to the translation initiating AUG, was replaced by C (mut5). The wild type and mutated constructs were transfected into 293T cells and their mRNAs analyzed by primer extension. Mutations within the motif from position 5 onward, including the single substitution of the central A (mut5), severely decreased transcription whereas mutations in the first four positions of the motif or in the sequence upstream to it had no significant effect (Fig. 7A). Thus the sequence necessary for transcription regulation lies in positions 5–11 of the motif, which are common to sequences important for translation initiation from short 5′UTR.

The first four nucleotides of the element, particularly those in positions 3 and 4, were shown to be important for YY1 binding and function [8], [9] but were not found necessary for TISU transcriptional activity. In addition, according to the transcription factor database (TRANSFAC 7.0) most of the functional YY1 binding sites are found at variable positions and orientations in promoters, raising the question whether the strictly localized and unidirectional TISU is a functional YY1 element. We therefore set out to determine which factor binds TISU. We employed the electrophoresis mobility shift assay (EMSA) using a radiolabeled oligonucleotide corresponding to the TISU sequence of PSMD8 as a probe and nuclear extract prepared from HeLa cells. The results show that TISU formed a single complex with the extract (Fig. 7B, lane 2). This complex was competed with by an excess of cold DNA that was used as a probe but not with an oligo corresponding to the Sp1 binding site (Fig. 7B, lanes 2, 3 and 6). The complex was not competed with by an oligo bearing a single A to C substitution (lane 4) but was efficiently competed with by an oligo containing the mutation in the first four nucleotides (mut2, lane 5). These findings are fully compatible with the functional analysis in which the A to C substitution, that diminished transcription also failed to bind TISU, while the first four nucleotides which were dispensable for TISU function, retained the binding activity. The results therefore strongly suggest that the protein(s) that binds TISU also mediates its transcription regulatory function. To test whether the protein that binds TISU is YY1 we added to the EMSA reactions YY1-specific antibodies or non-relevant control antibodies. As can be seen the YY1 antibodies supershifted the TISU complex whereas the control antibodies had no effect (Fig. 7B, lanes 7–8). Thus YY1 appears to be the major TISU binding protein in nuclear extract.

To analyze further the binding of YY1 to TISU, we performed competition assays with increasing amounts of a well-characterized and functional YY1 element from the c-myc gene [10]. As a control, equivalent amounts of either of cold PSMD8 TISU or the unrelated Sp1 oligos were used (Fig. 7C). The results clearly show that the c-myc YY1 site competed effectively with the TISU complex, whereas Sp1 failed to compete with this complex.

To examine the binding of YY1 to the PSMD8 promoter *in vivo*, we employed chromatin immunoprecipitation assays using antibodies against YY1 and non-relevant antibodies as a control. After reverse cross-linking semi-quantitative PCR reactions were performed with primers corresponding either to the proximal promoter region of PSMD8 or to the downstream coding region. As shown in Fig. 7D, YY1 is highly enriched on the PSMD8 promoter, but not in the downstream coding region. These results together suggest that YY1 mediates, at least in part, the function of TISU in transcription.

## Discussion

In this study we have characterized TISU as the first element operating both in translation initiation and transcription regulation. Using a computational search for over-represented proximal promoter motifs we identified TISU as an element found in ∼4% of mammalian genes, specifically located downstream to the TSS and highly enriched among genes with fundamental cellular functions such as mRNA and protein metabolisms. We demonstrated that TISU, which has an invariable ATG, composes a strong translation initiation context. Our detailed analysis of TISU function in translation established it as an element optimized to direct efficient translation initiation from mRNAs with an extremely short 5′UTR. Our findings characterized TISU as a novel translation initiator that is distinguished from the well-characterized Kozak element in its sequence and function. Positions −2 and −1 of TISU are distinct from those of the Kozak element and the nucleotide sequence in position +5 to +8 is unique to TISU and absent from the Kozak. Both the 5′ and the 3′ AUG flanking nucleotides cooperate to direct accurate and efficient translation initiation from short 5′UTR mRNAs. Considering the high translation fidelity from such short 5′UTRs, it remains to be seen whether or not this element directs initiation through the ribosome scanning mechanism.

TISU also plays a critical positive role in transcription. Our experiments suggest that the activity of TISU in transcription is mediated, at least in part, by the YY1 transcription factor. TISU's sequence is highly similar to the YY1 binding site and YY1 was found to be the major protein that binds TISU in nuclear extracts. Importantly, the effect of mutations in TISU on transcription fully correlates with YY1 binding activity, and YY1 occupies a TISU-containing promoter *in vivo*. The connection between transcription and the translational activity of the motif is highlighted by the finding that the same nucleotides (positions 5–12 of TISU) that are essential for transcription are also critical for the efficiency and fidelity of TISU activity in translation. However, positions 1–4 of TISU which appear to be important for translation, are dispensable for transcription and YY1 binding.

YY1 is a ubiquitously expressed transcription factor that plays crucial roles in various biological process including development, differentiation, cellular proliferation and apoptosis [11]. YY1 is a bifunctional regulatory factor that can either repress or activate transcription, depending on binding site context, protein interactions, or levels within the cell [12], [13], [14], [15], [16], [17]. Given the unique features of TISU that include strong positional and orientation bias and transcription and translation regulatory functions, it would be interesting to determine whether the duality in YY1 activity is also found in TISU genes.

In the fraction of genes in which TISU is present in the 5′UTR but does not compose the ORF initiation codon, its AUG is either out of frame with the downstream initiation codon or is followed by a stop codon (data not shown). Given the strong translation initiation capacity of TISU, it is likely that in these genes it competes with the downstream AUG, and behaves as a strong inhibitor of translation. We postulate that these genes should have a mechanism(s) that overcomes this inhibition, which would otherwise operate under certain conditions. As TISU could be a positive or negative translation regulatory element and YY1 can also be a positive or negative transcription regulatory factor, it is conceivable that different contexts of TISU can give rise to four combinations of transcription and translation modes of regulation (positive-positive; positive-negative; negative-positive; negative-negative) according to the physiological needs of the cell.

The present analysis of the proximal promoter enriched motif revealed a novel connection between transcription and translation initiation through a common regulatory element. Two other recent observations from our laboratory suggest that the influence of proximal promoter elements extends beyond the transcription initiation stage. In NF-κB-pathway regulated genes the core promoter type is linked to regulation of transcription elongation [18] and a genome wide bioinformatic analysis has revealed that core promoters are linked to the number and length of introns and to the lengths of 5′ and 3′ UTRs [3]. Our findings are an excellent basis for future studies aimed at characterizing the interplay between the transcription step and the succeeding stages of gene expression.

## Materials and Methods

### Bioinformatic analysis of the human proximal promoter

Human proximal promoter regions from −60 to +40 relative to the transcription start site (TSS) were retrieved from the EPD (http://www.epd.isb-sib.ch/) and the DBTSS (http://dbtss.hgc.jp/) and analyzed by the MEME (Multiple EM for Motif Elicitation) program [1], using the default parameters, searching for the most significant motifs of 6–12 nucleotides. For the gene functional annotation clustering, the Database for Annotation, Visualization and Integrated Discovery (DAVID), fifth version (http://david.abcc.ncifcrf.gov/gene2gene.jsp) was used, with the default parameters at medium classification stringency. To estimate the frequency of genes bearing TISU as translation initiator and those with a translation initiator downstream to the element, we retrieved the 5′UTR and the coding sequences of each gene from the UCSC Genome Browser (http://genome.ucsc.edu/). This information allowed us to determine the translation initiation site and the 5′UTR length. Occasionally there were inconsistencies between the TSSs assigned by the DBTSS and by the UCSC in which case the DBTSS site was chosen. In addition this data (11,120 genes) was used to retrieve the flanking sequence of the ORF's AUG from position −4 to +8 relative to the AUG. The genes were divided according to the 5′ flanking sequence of the AUG (AAS, RCC or neither) and according to their 5′ UTR length (short 5′ UTR: 4 to 45 nt ; long 5′ UTR: ≥46 nt). The sequences of each group were aligned to obtain sequence logo of the translation initiation site using WebLogo 3, version 2.8.2 (http://weblogo.berkeley.edu/).

### Plasmid construction

The promoter regions of the PSMD8 and WBP11 genes (from −180 to +50 and −150 to +50 respectively), were cloned by genomic PCR into pGL2-Basic (Promega) via SmaI and HindIII sites. Mutation of the whole TISU in both promoters was performed using a two-step PCR method. For the refined mutatgenesis of the TISU sequence in the PSMD8 promoter PCR was used, with oligonucleotides containing the mutated sequences flanked by a SacII site located immediately downstream to the TISU site. The PSMD8 and RPA39-EGFP constructs were prepared by removing the CMV promoter from EGFP-N1 (Clontech), using the AseI restriction enzyme and filling in with Klenow, and then digesting with HindIII. Then the promoter regions from −180 to +50 (PSMD8) and −150 to +47 (RPA39) were amplified and cloned in-frame to the EGFP ATG. The constructs used for *in vitro* transcription were prepared by substituting the CMV core promoter in the pEGFP-N1vector with the T7 promoter via ScaI and NheI sites. The ScaI site was inserted into the vector, using the Quikchange-Site Directed Mutagenesis kit (Stratagene), at position 538 to 543 upstream to the CMV TATA-box element. Oligonucleotides bearing TISU sequence and controls were cloned via Eco47III and Bgl II sites. Oligonucleotides bearing the TISU or Kozak sequence were inserted at NheI and BglII sites. These constructs were then used as templates to insert another 60 nt 5′UTR upstream to TISU and Kozak elements to increase the length of the 5′UTR (see Supporting Information S1 for primer description).

### Transient transfection assays and RNA analysis

293T cells were maintained and transfected as described [18]. 24 h after transfection total RNA was prepared using Tri-reagent (MRC Inc.). Primer extension was performed as previously described [19] using 20 µg of total RNA for either the luciferase primer or the EGFP primer and 2 µg RNA for the puro-GFP primer (supporting information S1). Primer extension of endogenous genes was performed using 10 to 20 µg total RNA prepared from non-transfected cells. The sequencing reaction was carried out with the Sequenase Version 2.0 kit (USB corporation). Results were visualized with a Phosphoimager (Fuji, BAS 2500). For RNA stability experiments cells were transfected with 50 ng of the WT or TISU mutant reporter genes and 24 hours later actinomycin D (10 µg/ml) was added. RNA was extracted at different time points using the RNeasy kit (Qiagen) and the mRNA level analyzed by RT-PCR with 5′ primers specific to each construct (supporting information S1).

### Electrophoretic mobility shift assay

Oligonucleotides used as probes were end-labeled using T4 PNK (Fermentas) and then annealed. Binding reactions were performed in a buffer containing 25 mM HEPES (pH 7.9), 50 mM KCl, 1 mM DTT, 10% glycerol, 2 µg of poly(dI-dC) and 2 µg of HeLa nuclear extract prepared as described previously [20]. The reaction mix was incubated on ice for 10 min after which 50 fmole probe was added for an additional 20 min. Competitor DNAs were added prior to the addition of the probe. In the super-shift reactions 400 µg of YY1 antibodies (SantaCruz, C20) were added to the primary mix and incubated for 15 min at RT. Then the probe was added and the mix incubated on ice for an additional 20 min. The reactions were separated by native eloctrophoresis at 4°C in a 4.87% polyacryamide gel with 1× Tris-Glycine buffer at 185 V. The gel was dried and results were visualized with a Phosphoimager (Fuji, BAS 2500).

### In vitro transcription and translation

The constructs containing the T7 promoter were linearized by AflII or prepared by PCR. Capped mRNA was synthesized using RiboMAX™ Large Scale RNA Production Systems-SP6 and T7 (Promega) with the addition of Ribo m^7^G Cap Analog (Promega). RQ1 RNase-Free DNaseI was added, the mRNA extracted with phenol∶chloroform and precipitated with ethanol. The capped mRNAs were denatured at 65°C for 10 min and then placed on ice for 2 min. 1 µg of each mRNA was used for *in vitro* translation with TNT Coupled Reticulocyte Lysate Systems (Promega) with the addition of ^35^S methionine. Then 10% of each reaction was loaded onto 15% PAGE. For the *in vivo* translation assay 10 µg of the *in vitro* transcribed mRNA and 5 µg of luciferase mRNA, as internal control, were denatured and co-transfected into 293T cells which had been previously seeded on 12-well plates, using 15 µg Lipofectamine Reagent (Invitrogen). 24 hours after transfection total cell extracts were prepared. Transfection efficiency was normalized by measuring luciferase activity and normalized extracts were then subjected to western blot using anti-GFP mAb.

### Chromatin Immunoprecipitation assay

Chromatin extract from Hela cells was prepared as described previously [21], then immunoprecipitated with YY1 antibodies or irrelevant antibodies as control. The precipitated fragments were quantified by PCR using primers for the promoter and the coding region of PSMD8 (see supporting information S1 for primer description).

## Supporting Information

Supporting Information S1 Primer list
(0.05 MB DOC)

## References

[1] Elkan TLBaC (1994).

[2] Schug J, Schuller WP, Kappen C, Salbaum JM, Bucan M (2005). Promoter features related to tissue specificity as measured by Shannon entropy.. Genome Biol.

[3] Moshonov S, Elfakess R, Golan-Mashiach M, Sinvani H, Dikstein R (2008). Links between core promoter and basic gene features influence gene expression.. BMC Genomics.

[4] Kozak M (1999). Initiation of translation in prokaryotes and eukaryotes.. Gene.

[5] Kozak M (1994). Determinants of translational fidelity and efficiency in vertebrate mRNAs.. Biochimie.

[6] Kozak M (1997). Recognition of AUG and alternative initiator codons is augmented by G in position +4 but is not generally affected by the nucleotides in positions +5 and +6.. Embo J.

[7] Kozak M (1991). Structural features in eukaryotic mRNAs that modulate the initiation of translation.. J Biol Chem.

[8] Johansson E, Hjortsberg K, Thelander L (1998). Two YY-1-binding proximal elements regulate the promoter strength of the TATA-less mouse ribonucleotide reductase R1 gene.. J Biol Chem.

[9] Satyamoorthy K, Park K, Atchison ML, Howe CC (1993). The intracisternal A-particle upstream element interacts with transcription factor YY1 to activate transcription: pleiotropic effects of YY1 on distinct DNA promoter elements.. Mol Cell Biol.

[10] Riggs KJ, Merrell KT, Wilson G, Calame K (1991). Common factor 1 is a transcriptional activator which binds in the c-myc promoter, the skeletal alpha-actin promoter, and the immunoglobulin heavy-chain enhancer.. Mol Cell Biol.

[11] Gordon S, Akopyan G, Garban H, Bonavida B (2006). Transcription factor YY1: structure, function, and therapeutic implications in cancer biology.. Oncogene.

[12] Bushmeyer S, Park K, Atchison ML (1995). Characterization of functional domains within the multifunctional transcription factor, YY1.. J Biol Chem.

[13] Lee JS, See RH, Galvin KM, Wang J, Shi Y (1995). Functional interactions between YY1 and adenovirus E1A.. Nucleic Acids Res.

[14] Lee YM, Lee SC (1994). Transcriptional activation of the alpha-1 acid glycoprotein gene by YY1 is mediated by its functional interaction with a negative transcription factor.. DNA Cell Biol.

[15] Park K, Atchison ML (1991). Isolation of a candidate repressor/activator, NF-E1 (YY-1, delta), that binds to the immunoglobulin kappa 3′ enhancer and the immunoglobulin heavy-chain mu E1 site.. Proc Natl Acad Sci U S A.

[16] Seto E, Shi Y, Shenk T (1991). YY1 is an initiator sequence-binding protein that directs and activates transcription *in vitro*.. Nature.

[17] Shi Y, Seto E, Chang LS, Shenk T (1991). Transcriptional repression by YY1, a human GLI-Kruppel-related protein, and relief of repression by adenovirus E1A protein.. Cell.

[18] Amir-Zilberstein L, Ainbinder E, Toube L, Yamaguchi Y, Handa H (2007). Differential regulation of NF-kappaB by elongation factors is determined by core promoter type.. Mol Cell Biol.

[19] Ainbinder E, Amir-Zilberstein L, Yamaguchi Y, Handa H, Dikstein R (2004). Elongation inhibition by DRB sensitivity-inducing factor is regulated by the A20 promoter via a novel negative element and NF-kappaB.. Mol Cell Biol.

[20] Amir-Zilberstein L, Dikstein R (2008). Interplay between E-box and NF-kappaB in regulation of A20 gene by DRB sensitivity-inducing factor (DSIF).. J Biol Chem.

[21] Ainbinder E, Revach M, Wolstein O, Moshonov S, Diamant N (2002). Mechanism of rapid transcriptional induction of tumor necrosis factor alpha-responsive genes by NF-kappaB.. Mol Cell Biol.

