# Supplementary material for: A Translation Initiation Element Specific to mRNAs with Very Short 5′UTR that Also Regulates Transcription
Source: PLoS One. 2008 Aug 28;3(8):e3094. doi: 10.1371/journal.pone.0003094 (PMC2518114; doi:10.1371/journal.pone.0003094)
Supplement: Supporting Information S1 — Primer list (0.05 MB DOC) [file pone.0003094.s001.doc]

**Supporting information S1**

**Primers list**

*Primers used for cloning promoters into pGL2-Basic and for performing the two steps PCR mutagenesis in TISU*:

PSMD8 forward: 5’ ttcattaagggcagggctcc.

PSMD8 reverse (Hind III): 5’ cccccAAGCTTggctcgagaagcctgcc.

PSMD8 mut Step 1(f1) reverse: 5' gctCCTCTTCCCTTCaaaccTcgcgcccctccg.

PSMD8 mut Step 1(f2) forward: 5' GAAGGGAAGAGGcgcggcggtgaacgggg.

WBP11 forward: 5’ ccgacaaccaaccaattgag.

WBP11 reverse (HindIII): 5’ cccccAAGCTTttcaactgggtctctcggtc.

WBP11 mut step 1(f1) reverse: 5' gctCCTCTTCCCTTCaaaccTcgcgcccctccg.

WBP11 mut Step 1(f2) forward: 5' GAAGGGAAGAGGagctgaggggttgaccgag.

RPA39 mut Step 1(f1) reverse: 5’ CTGAGAACCTCTTCCCTTCAATCTCTCCACGAGAC

RPA39 mut Step 1(f2) forward: 5’ GAAGGGAAGAGGTTCTCAGGCGGTGGAGG

*Primers used for performing the serious of mutations in TISU within PSMD8 promoter*:

PSMD8 forward: 5’ ttcattaagggcagggctcc.

PSMD8 mut1 reverse (SacII): 5’ tcaccgccgcggccgccatcttgcCACTtgcggcaagcagaccgc.

PSMD8 mut2 reverse (SacII): 5’ tcaccgccgcggccgccatTGAAcgtgatgcggcaagcagac.

PSMD8 mut3 reverse (SacII): 5’ tcaccgccgcggccgcAGGcttgcgtgatgcggcaagc.

PSMD8 mut4 reverse (SacII): 5’ tcaccgccgcggATCTcatcttgcgtgatgcggc.

PSMD8 mut5 reverse (SacII): 5’ tcaccgccgcggccgccaGcttgcgtgatgcggcaag.

*Primers used for cloning the PSMD8 and RPA39 promoters into pEGFP-N1:*

PSMD8 forward: 5’ ttcattaagggcagggctcc.

PSMD8 reverse (HindIII): 5’ CCCCCAAGCTTGCTCGAGAAGCCTGCCGC.

RPA39 forward: 5’ TTGGACAAAAGCAACTTCCG.

RPA39 reverse (HindIII): 5’ CCCCCAAGCTTGCTCCGCATTTCCTCCAC.

*Primers used to the insertion of ScaI site into pEGFP-N1 using site directed mutagenesis:*

ScaI forward: 5’ GGGCGGTAGGCGAGTACTGTGGGAGGTC.

ScaI reverse: 5’ GACCTCCCACAGTACTCGCCTACCGCCC.

*Primers used to estimate the function of TISU as a translational initiation elemen:*

TISU forward: 5’ CAAGATGGCGGCA.

TISU reverse: 5’ GATCTGCCGCCATCTTG.

Random sequence forward: 5’ GGGAAGCTGGAGA.

Random sequence reverse: 5’ GATCTCTCCAGCTTCCC.

Random sequence +ATG forward: 5’ GGGAATGTGGAGA.

Random sequence +ATG reverse: 5’ GATCTCTCCACATTCCC.

Primer to generate the 100bp forward (XbaI): 5’ CCCCCTCTAGAAATAATTTTCTGGATTATTG.

Primer to generate the 100bp reverse (NheI): 5’ CCCCCGCTAGCGAATTTTTCGCAGCCTACCG.

T7 promoter forward: 5’ GTAATACGACTCACTATAGGG.

T7 promoter reverse: 5’ CTAGCCCTATAGTGAGTCGTATTAC.

TISUmut1 forward: 5’ CTAGCTTCAATGGCGGCA.

TISUmut1 reverse: 5’ GATCTGCCGCCATTGAAG.

TISUmut2 forward: 5’ ctagccaagatgagatca.

TISUmut2 reverse: 5’ GATCTGATCTCATCTTGG.

TISUmut3 forward: 5’ CTAGCTACAATGGCGGCA.

TISUmut3 reverse: 5’ GATCTGCCGCCATTGTAG.

TISUmut4 forward: 5’ ctagccaagatgGgatca.

TISUmut4 reverse: 5’ GATCTGATCCCATCTTGG.

TISUmut5 forward: 5’ CTAGCCATAATGGCGGCA.

TISUmut5 reverse: 5’ GATCTGCCGCCATTATGG.

TISU to Kozak forward: 5’ CTAGTACAATGGGATCA

TISU to Kozak reverse: 5’ GATCTGATCCCATTGTAG.

Kozak RCCATGG forward: 5’ ctagcgaccatggggaga.

Kozak RCCATGG reverse: 5’ GATCTCTCCCCATGGTCG.

Kozak_RCCRCCATGG forward: 5’ CTAGCGCCACCATGGGGAGA.

Kozak_RCCRCCATGG reverse: 5’ GATCTCTCCCCATGGTGGCG.

Primer used to add a 60 nt 5’UTR long:

5’ GTAATACGACTCACTATAGGGCAACCCCTTTTTGGAAAC.

Primer located downstream to the EGFP polyA signal used to prepare the templates for the *in vitro* transcription reaction, reverse: 5’ CGCCTTAAGATACATTGATG.

*Primers used for the semi-quantitative PCR to measure the effect TISU on mRNA stability within PSMD8 promoter:*

PSMD8 wt forward: 5’ TTGCCGCATCACGCAAGATG.

PSMD8 mut forward: TTGCCGCATCACGGAAGGGA.

Luc Bot2 reverse: CCAGGAACCAGGGCGTATCT.

*Primers used for primer extension:*

+109 Luc: 5’ GCCTTTCTTTATGTTTTTGGCG.

+66 pEGFP-puro: 5’ TGTACTCGGTCATGGTAAGC. (Internal control)

PSMD8 -109: 5’ TTGCTCGTACATGCCGGTCG.

WBP11 -122: 5’ TTCTGCTGTGCTCCCAGGAC.

EGFP -1 (figure 6): 5’ ATGGTGGCGACCGGTGGAT.

*Oligos used for Electrophoretic Mobility Shift Assay:*

YY1 wt forward: 5’ ATCACGCAAGATGGCGGCCGCGG.

YY1 wt reverse: 5’ CCGCGGCCGCCATCTTGCGTGAT.

YY1 mut2 forward: 5’ ATCACGTTCAATGGCGGCCGCGG.

YY1 mut2 reverse: 5’ CCGCGGCCGCCATTGAACGTGAT.

YY1 mut5 forward: 5’ ATCACGCAAGCTGGCGGCCGCGG.

YY1 mut5 reverse: 5’ CCGCGGCCGCCAGCTTGCGTGAT.

Sp1 forward: 5’ ATTCGATCGGGGCGGGGCGAGCT.

Sp1 reverse: 5’ AGCTCGCCCCGCCCCGATCGAAT.

YY1 of c-myc forward: 5’GAAGAGAAAATGGTCGGGCGCGCG.

YY1 of c-myc reverse: 5’ CGCGCGCCCGACCATTTTCTCTTC.

*Primers used for the semi-quantitative PCR of the ChIP assay:*

PSMD8 promoter forward: 5’ GGGCTGAGGCAGGTTGTAG.

PSMD8 promoter reverse: 5’ Gttccactcgcccttgagt.

PSMD8 coding forward: 5’ tttccaagtgggaagaccag.

PSMD8 coding reverse: 5’ ccaccagtgctaccattcc.
